# Physician and Trainee Perceptions of Telecritical Care Practice and Education: Results of a Programmatic Survey

**DOI:** 10.1089/tmr.2022.0037

**Published:** 2022-12-26

**Authors:** Milad Sharifpour, Timothy Buchman, Cheryl Hiddleson, Craig S. Jabaley, Jayashree Raikhelkar

**Affiliations:** ^1^ Department of Anesthesiology, Cedars-Sinai Medical Center, Los Angeles, California, USA.; ^2^ Department of Surgery, Electronic ICU Service, Emory Healthcare, Atlanta, Georgia, USA.; ^3^ Department of Surgery, Emory Critical Care Center, Atlanta, Georgia, USA.; ^4^ Emory Healthcare, Atlanta, Georgia, USA.; ^5^ Department of Anesthesiology, Emory University School of Medicine, Atlanta, Georgia, USA.; ^6^ TeleCritical Care East, Veterans Health Administration, Cincinnati, Ohio, USA.

**Keywords:** education, telecritical care, telemedicine, trainee perceptions

## Abstract

**Background:**

Telecritical care (TCC) as a telehealth modality seeks to remedy contemporary shortfalls in staffing and experience at the bedside. Physician and physician trainee perceptions of TCC practice and education can help inform programmatic and curricular decisions. The perceptions of TCC and a formalized structured TCC rotation from faculty and trainees are unknown.

**Objective:**

To evaluate perceptions of TCC practice and education among participating physicians and trainees.

**Methods:**

Survey of physicians and trainees participating in the Emory Critical Care Center's TCC unit from 2017 to 2021 was conducted, after implementation of a structured TCC educational curriculum. Items were developed with a 5-point Likert scale.

**Results:**

The overall response rate was 71% (43 of 61). Most respondents felt their knowledge was used appropriately and that their recommendations were well received at the bedside. The majority perceived that the TCC program improved continuity, quality, and safety of patient care. More than half of respondents would practice TCC in the future, and most would advocate for it. Most fellows were comfortable providing patient care remotely after their rotation. The majority of respondents felt TCC did not add to their level of burnout.

**Conclusions:**

This programmatic evaluation identified perceived improvements in patient care. Implementation of a TCC rotation does not seem to negatively impact the educational experience of trainees.

## Introduction

Telecritical care (TCC) is a modality of critical care delivery addressing the intensivist shortage in the United States.^1,2^ Survey studies of bedside staff, physicians, and trainees with TCC support have indicated a high level of acceptance of this technology.^3–5^ However, the views of those in training, and of those practicing TCC remain unknown. The objective of this program evaluation was to examine the perception of TCC's impact on patient care among TCC trainees and practitioners, and their perception regarding a formalized TCC educational curriculum.

## Methods

We surveyed multidisciplinary critical care fellows and faculty rotating through Emory Critical Care Center's (ECCC) electronic intensive care unit (EICU) from initiation of elective TCC rotation for fellows in January 2017 until July 2021. The program at the time supported 160 intensive care unit (ICU) beds and 12 emergency department beds at affiliated hospitals and unaffiliated community sites. EICU provides continuous TCC nursing support with periodic rounding and triage, smart alarm recognition and intervention, facilitation of evidence-based practice, and mentoring of new/inexperienced bedside staff.

EICU provides teleintensivist coverage from 7 pm to 7 am at night, and daytime coverage from 7 am to 7 pm on weekends and holidays. After 7 pm, bedside advanced practice providers are supported by the teleintensivist using a tele-ICU system (eCaremanager; Phillips Corporation, Cambridge, MA).

In 2018–2019, 6 of the 31 fellows completed the rotation with an Emory attending physician in Royal Perth Hospital, in Perth, Western Australia, which has a 12-h time difference with Atlanta during eastern daylight time. These fellows worked during the daytime (7 am to 7 pm) to provide care at night remotely to hospitals covered by the Emory EICU in Georgia, USA.

Average duration of the rotation for fellows working in Atlanta was 2 weeks, and 1 month for those working in Perth. At the start of the rotation, each fellow received a checklist-driven orientation guided by an experienced EICU attending (Table 1). Fellows practiced with graduated autonomy and were responsible for all aspects of care, including remote patient assessment, communication with bedside staff and families, as well as the generation of clinical documentation.

**Table 1.** Emory Electronic Intensive Care Unit Orientation Checklist

Clinical learning/training:
1. Opening of workstation at start of shift
2. Understanding eCare manager: a. Video assessment b. Waveform display c. eLert process/smart alerts
3. Organization of workstation
4. Admission note writing outline: a. Expanded EICU physician note b. Brief progress note c. Comprehensive progress note
5. Video assessment etiquette
6. TCC intraunit communication (provider and RN)
7. Emory EICU protocols: a. Admission to ICU process b. Clinical assessment process c. Provider presence at bedside d. Cardiopulmonary resuscitation e. Family communication workflow EICU specific policies
8. Billing: a. billing b. Recording of billing within EICU note
9. Workstation cleanliness/virus prevention
10. Location of hospital-specific documents: worksheets, faxes, order sets, important phone numbers, affiliate schedules
11. Categories I, II, and III physician limitations/communication
12. Closure of workstation

EICU, electronic intensive care unit; ICU, intensive care unit; RN, registered nurse; TCC, telecritical care.

Two survey instruments, one for fellows and another for faculty, were designed by the authors, who all are clinician educators and there was no further testing done. The 2 instruments shared 11 common items with 2 additional items addressed to the trainees (*N* = 13 total) and 1 additional for the physicians (*N* = 12; Table 2). Each item was developed with response ranking per a 5-point Likert scale. Fellows reported their postgraduate year, and faculty reported their years of TCC experience. Table 3 provides a breakdown of the fellows' answers with regard to the location they completed the rotation (Atlanta or Perth).

**Table 2.** Emory Electronic Intensive Care Unit Survey

Fellow PGY: range 4–10; mean 5.7; median 5; mode 5 Attending TCC experience (years): range 1–7; mean 4.6, median 5, mode 6	Total number of respondents (T): 43 fellows (F): 24 attendings (A): 19						
	**Strongly agree**	**Agree**	**Neutral**	**Disagree**	**Strongly disagree**	***R***	***p***
1. While practicing TCC, I felt my knowledge and skills in critical care were well utilized	F (9)	F (12)	F (1)	F (1)	F (1)	0.04	0.75
	A (8)	A (9)	A (1)		A (1)		
	T = 17	T = 21	T = 2	T = 1	T = 2		
2. While on a shift, my clinical recommendations were followed by bedside providers and staff	F (10)	F (8)	F (5)		F (1)	0.002	1
	A (6)	A (11)	A (1)	A (1)			
	T = 16	T = 19	T = 6	T = 1	T = 1		
3. The input of the tele-ICU team (attending, trainees, nurses) improved the continuity of patient care	F (10)	F (9)	F (2)	F (1)	F (2)	0.04	0.75
	A (6)	A (9)	A (4)				
	T = 16	T = 18	T = 6	T = 1	T = 2		
4. The input of the tele-ICU team improved the quality of patient care	F (10)	F (10)	F (3)		F (1)	0.01	0.93
	A (8)	A (8)	A (3)				
	T = 18	T = 18	T = 6		T = 1		
5. The input of the tele-ICU team improved the safety of patient care	F (11)	F (10)	F (2)		F (1)	0.03	0.82
	A (7)	A (11)	A (1)				
	T = 18	T = 21	T = 3		T = 1		
6. Interacting with tele-ICU improved patient and family satisfaction	F (2)	F (9)	F (10)	F (2)	F (1)	0.004	0.98
	A (3)	A (4)	A (11)	A (1)			
	T = 5	T = 13	T = 21	T = 3	T = 1		
7. The tele-ICU rotation represents a valuable educational experience	F (12)	F (10)	F (1)		F (1)	0.16	0.29
	A (7)	A (8)	A (4)				
	T = 19	T = 18	T = 5		T = 1		
8. I experienced burnout during the rotation/shift	F (1)		F (3)	F (6)	F (14)	0.6	0.00
	A (4)	A (2)	A (8)	A (4)	A (1)		
	T = 5	T = 2	T = 11	T = 10	T = 15		
9. I felt Emory's approach to critical care is innovative	F (8)	F (11)	F (4)		F (1)	0.07	0.64
	A (6)	A (7)	A (5)	A (1)			
	T = 14	T = 18	T = 9	T = 1	T = 1		
10. After working in the EICU, I would be more likely to practice TCC in the future.	F (8)	F (6)	F (6)	F (3)	F (1)	0.18	0.24
	A (2)	A (7)	A (6)	A (3)	A (1)		
	T = 10	T = 13	T = 12	T = 6	T = 2		
11. After working in the EICU, I would be more likely to advocate for TCC in my future institutions of work	F (6)	F (10)	F (4)	F (3)	F (1)	0.19	0.20
	A (8)	A (7)	A (3)		A (1)		
	T = 14	T = 17	T = 7	T = 3	T = 2		
Additional question asked to faculty (*N* = 19)							
I feel the fellows' EICU rotation is an essential rotation for future practice as an attending in TCC.	T = 6	T = 7	T = 4	T = 2			
Additional questions asked to fellows (*N* = 24)							
After completing the rotation, I am comfortable managing unstable critically ill patients virtually	T = 9	T = 9	T = 3	T = 2	T = 1		
After completing the rotation, I am comfortable using TCC software that allows me to visualize and communicate with patients and bedside staff	T = 10	T = 12		T = 1	T = 1		

PGY, postgraduate year.

**Table 3.** Emory Electronic Intensive Care Unit Fellows' Survey

Respondents: 24 Location: Atlanta (A): 18 Perth (P): 6	Strongly agree	Agree	Neutral	Disagree	Strongly disagree	***R***	***p***
1. During the tele-ICU rotation, I felt my knowledge and skills in critical care were well utilized	P (3)	P (2)		P (1)		0.09	0.68
	A (6)	A (10)	A (1)		A (1)		
	T = 9	T = 12	T = 1	T = 1	T = 1		
2. While on the rotation, my clinical recommendations were followed by bedside providers and staff	P (3)	P (1)	P (2)			0.02	0.91
	A (7)	A (7)	A (3)		A (1)		
	T = 10	T = 8	T = 5		T = 1		
3. The input of the tele-ICU team (attending, trainees, nurses) improved the continuity of patient care	P (3)	P (1)	P (1)		P (1)	0.007	1
	A (7)	A (8)	A (1)	A (1)	A (1)		
	T = 10	T = 9	T = 2	T = 1	T = 2		
4. The input of the tele-ICU team improved the quality of patient care	P (3)	P (1)	P (2)			0.01	0.97
	A (7)	A (9)	A (1)		A (1)		
	T = 10	T = 10	T = 3		T = 1		
5. The input of the tele-ICU team improved the safety of patient care	P (3)	P (2)	P (1)			0.02	0.94
	A (8)	A (8)	A (1)		A (1)		
	T = 11	T = 10	T = 2		T = 1		
6. Interacting with tele-ICU improved patient and family satisfaction		P (2)	P (3)		P (1)	0.17	0.41
	A (2)	A (7)	A (7)	A (1)	A (1)		
	T = 2	T = 9	T = 10	T = 1	T = 2		
7. After completing the rotation, I am comfortable managing unstable critically ill patients virtually	P (3)	P (2)	P (1)			0.16	0.43
	A (6)	A (7)	A (3)	A (1)	A (1)		
	T = 9	T = 9	T = 4	T = 1	T = 1		
8. After completing the rotation, I am comfortable using TCC software	P (3)	P (3)				0.13	0.52
	A (7)	A (9)		A (1)	A (1)		
	T = 10	T = 12		T = 1	T = 1		
9. The tele-ICU rotation represents a valuable educational experience	P (4)	P (2)				0.21	0.31
	A (8)	A (8)	A (1)		A (1)		
	T = 12	T = 10	T = 1		T = 1		
10. I experienced burnout during the rotation				P (2)	P (4)	0.15	0.47
	A (1)		A (3)	A (4)	A (10)		
	T = 1		T = 3	T = 6	T = 14		
11. I felt Emory's approach to critical care is innovative	P (2)	P (3)	P (1)			0.03	0.88
	A (6)	A (8)	A (3)		A (1)		
	T = 8	T = 11	T = 4		T = 1		
12. After the rotation, I would be more likely to practice TCC as an attending in the future	P (3)		P (1)	P (2)		0.0	1
	A (5)	A (6)	A (5)	A (1)	A (1)		
	T = 8	T = 6	T = 6	T = 3	T = 1		
13. After this rotation, I would be more likely to advocate for TCC in my future institutions of work	P (1)	P (2)	P (1)	P (2)		0.2	0.34
	A (5)	A (8)	A (3)	A (1)	A (1)		
	T = 6	T = 10	T = 4	T = 3	T = 1		

Survey responses were considered nonparametric ordinal categorical variables for the purposes of analysis. Responses by respondent category were considered independent samples and compared on a per-question basis using the Wilcoxon rank sum test (i.e., Mann–Whitney test). Results are reported with corresponding test effect size estimates (*R* values). *p*-Values <0.05 were considered statistically significant and indicative of discordant responses between the respondent categories. Participation was voluntary with surveys e-mailed to all EICU faculty and fellows. Reminder e-mails were sent weekly for 4 weeks. We report our results as item-level frequencies.

## Results

The faculty and fellow response rates were 63% (19/30) and 77% (24/31), respectively.

The combined response rate was 70.5% (43/61). The primary specialties of the 24 responding TCC fellows are provided in Supplementary Figure S1. The results of the paired questionnaire are presented in Table 1. Most respondents felt their skills and knowledge were well utilized [88% (38/43)] and their recommendations were followed by the bedside providers and staff [81% (35/43)]. Most perceived the tele-ICU team improved the continuity, quality, and safety of patient care. Forty-nine percent (21/43) of respondents were neutral, and 42% (18/43) agreed that TCC improved patient and family satisfaction. Eighty-six percent (37/43) agreed that the rotation was a valuable experience for fellows.

More than two-third of faculty (13/19) felt that providing TCC support did not add to their level of burnout, and 83% (20/24) of fellows did not experience burnout during the rotation. More than half of fellows were likely to practice TCC in the future, and >70% total respondents (31/43) would advocate for TCC implementation in their future institutions of work. Sixty-eight percent (13/19) of the faculty felt that the EICU rotation is an essential rotation in critical care fellowship. More than 75% (18/24) of the fellows agreed they were comfortable with the TCC technology and managing critically ill patients remotely. When comparing perceptions of fellows completing the rotation in Perth to Atlanta, the proportions of responses were similar as presented in Table 2.

## Discussion

Our program evaluation showed that TCC has perceived improvements in patient care with the majority of faculty and trainees not perceiving burnout issues. Implementation of a TCC rotation in our critical care fellowship does not seem to negatively impact the critical care educational experience of trainees.

There are limited data regarding perceptions of TCC practitioners and fellows in training. Owing to lost educational opportunities in medical education and residency during the COVID-19 pandemic,^6^ a few programs leveraged existing TCC infrastructure to develop clinical rotations for medical students and residents to provide critical care skills and education in a safe environment with positive results.^7–9^

In our survey, there appeared to be a strong sense among faculty and fellows that TCC improved patient care, especially its quality, continuity, and safety. This has been replicated in other surveys with residents interacting with TCC services.^5–10^ Very few faculty and trainees felt TCC worsens patient and family satisfaction. This was contrary to a TCC survey examining perceptions of resident trainees receiving TCC services.^5^ The majority of the faculty and fellows felt working in our TCC unit did not add to their level of burnout despite working unsocial hours.

Interestingly, fellows working night-time shifts in Atlanta did not report experiencing more burnout than their counterparts in Perth. Burnout is common among critical care clinicians, and its prevalence has increased during the COVID-19 pandemic.^11,12^ Implementation of a TCC service may reduce burnout among brick-and-mortar ICU providers by allowing for uninterrupted sleep and improved quality of life.^13^ One explanation for these results could be that all faculty were surveyed during the COVID-19 pandemic, and TCC may have allowed for patient management and communication with bedside staff and families without the constant worry for personal safety.

Our survey has limitations. This program evaluation was conducted in a single academic institution. Our questionnaire pertains to practicing the traditional hub and spoke model of TCC and not other models of TCC. Another point to note is that most faculty also practice in brick-and-mortar ICUs covered by the ECCC EICU. Their pre-existing relationships with bedside staff may make their interactions as TCC providers more seamless. A response rate of 71% raises the possibility of nonresponse bias that may limit the conclusions drawn.

The next steps would be to measure quality indicators in the ICU with TCC support, and the degree of burnout among faculty and the educational impact of the TCC rotation on trainees.

## Conclusion

Our program evaluation, which is the first to evaluate the perceptions of TCC faculty and fellows, suggests that in our practice of TCC, there are perceived improvements in patient care with limited levels of burnout experienced by TCC practitioners. Implementation of a TCC rotation in critical care fellowship does not seem to negatively impact the critical care educational experience of trainees.

## Supplementary Material

Supplemental data

## Abbreviations Used

ECCC: Emory Critical Care Center
EICU: electronic intensive care unit
ICU: intensive care unit
PGY: postgraduate year
TCC: telecritical care
