# Supplementary material for: Physician and Trainee Perceptions of Telecritical Care Practice and Education: Results of a Programmatic Survey
Source: Telemed Rep. 2022 Dec 26;3(1):201–5. doi: 10.1089/tmr.2022.0037 (PMC9811849; doi:10.1089/tmr.2022.0037)

Supplement 1: Primary Speciality of TCC Fellows

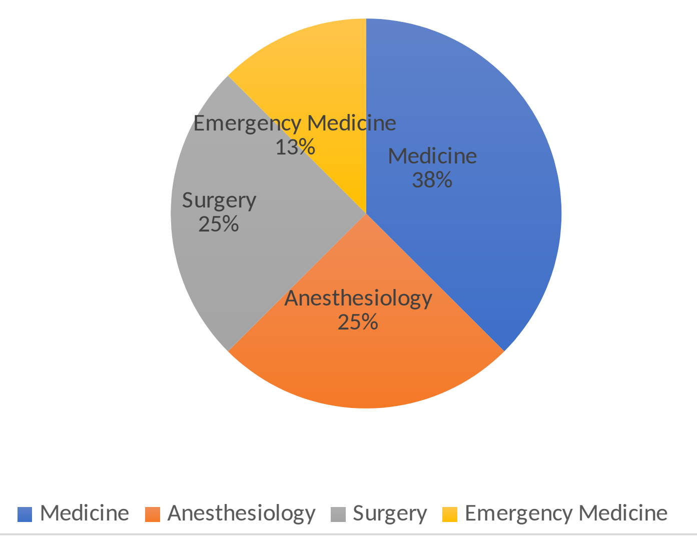

Supplement: Supplementary file 1 [file Suppl_FigS1.docx]
